# 

**Published:** 1874-10

**Authors:** 


### CONTENTS:

ORIGINAL COMMUNICATIONS:

ART. I. Typhoid Fever. Collation of 112 Cases. By W. T. Montgomery, M.D ........ 577
ART. II. Case of Vesico-Vaginal Fistula. By J. E. O'Brien, M.D.... .. 591
ART. III. "Infants and their Food." The Reviews—Summary ........ 594
ART. IV. Thoughts suggested by a perusal of Dr. Whitmire's Paper on Criminal Abortion, etc. By G. W. Emery, M.D. ........ 598

PROGRESS IN MEDICAL SCIENCES:

ART. I. Progress in Obstetrics and Gynæcology. By Dr. A. R. Jackson. 600
ART. II. Progress of Surgery. By Dr. J. E. Owens. ........ 605
ART. III. Progress in the Practice of Medicine. By Dr. N. Bridge.... 611

REPORTS OF SOCIETIES ........ 615
CLINICS ........ 623
HOSPITALS, ........ 625
EDITORS' BOOK TABLE ........ 627
EDITORIAL ........ 631
MORTALITY STATISTICS ........ 638

